# Identification of troponin I and actin, alpha cardiac muscle 1 as potential biomarkers for hearts of electrically stimulated chickens

**DOI:** 10.1186/1477-5956-10-1

**Published:** 2012-01-10

**Authors:** Azura Amid, Norshahida A Samah, Faridah Yusof

**Affiliations:** 1 Bioprocess and Molecular Engineering Research Unit, Faculty of Engineering, International Islamic University Malaysia, P.O. Box 10, 50728 Kuala Lumpur, Malaysia; 2 Nanoscience and Nanotechnology Research Group, Department of Biotechnology Engineering, International Islamic University Malaysia, P.O. Box 10, 50728 Kuala Lumpur, Malaysia

**Keywords:** Actin, alpha cardiac muscle, stunning, slaughtering, proteomics, troponin I

## Abstract

**Methods:**

In this study, proteomics methods have been used to study the effects of different currents and voltages used to stun chickens. Protein profiles of chicken hearts were constructed to detect differences in protein expression and modification. The different voltages studied were 10 V, 40 V and 70 V, while the currents examined were 0.25 A, 0.5 A, and 0.75 A. The profiles obtained from these stunning conditions were compared to the non-stunned (0 A, 0 V) sample.

**Results:**

Proteomics analyses using 2D Platinum ImageMaster 6.0 and Matrix-assisted laser desorption/ionization with time-of-flight mass spectrometry (MALDI-TOF) identified troponin I and alpha cardiac muscle actin 1 in the electrically stimulated heart samples. The overexpression of the proteins was further confirmed at the transcriptional level by Real Time PCR.

**Conclusion:**

The results from MALDI-TOF and Real Time PCR agreed; therefore, this method for identifying biomarkers of electrically stimulated chicken hearts provides a novel approach for differentiation the hearts of increased electrically stimulated chickens from those of non-stunned chickens.

## Background

Stunning is used to induce unconsciousness in animals prior to exsanguinations and slaughter. Standards for stunning may vary from one country to another. In certain countries, such as Malaysia, the standard for stunning is minimized because animals must be alive during the slaughtering process to meet the Muslim requirements for food production. The same situation is also experienced in kosher food production. Stunning by high voltage and current may introduce cardiac fibrillation in animals and lead to death; therefore, the need to study the effect of an increased current and voltage on biological systems during the stunning procedure has arisen. Unlike DNA, which is stable and static, protein expression is dynamic and influenced by both internal and external stimuli. In this study, immobilization was considered to be one type of external stimulus that excites the expression and regulation of proteins in electrically stimulated chickens.

Proteomics is a widely used tool in biomarker identification, especially in studies of disease development and cancer. A biomarker is a substance found in the blood, urine, cerebrospinal fluid, or tissues and is often detected in abnormally high amounts in samples from animals with certain diseases or conditions [1]. For many diseases, such as cancer, protein function is altered in the context of key signaling pathways that regulate critical cellular functions, including proliferation, apoptosis, differentiation, survival, immunity, metabolism, invasion, and metastasis [2]. The validation of a biomarker requires the analysis of thousands of samples to ensure that the potential biomarker is indeed related to a disease state and is not simply a function of the variability among samples due to differences in diet, genetic background, lifestyle, and so on [3,4]. Although biomarker discovery has been mainly focused on prognosis and diagnosis of a disease, this approach is also suitable for other applications, such as identifying altered proteins in chickens during stunning and slaughter.

Electrical stunning is accomplished by passing a sufficient amount of electrical current through the brain of a bird for a given amount of time. Electrical stunning methods used in the past include electrified knives [5], electrified contact grids or plates and a V-shaped stunner, but the most common is the water bath stunner. The electrical current passes through the body from the head, which is in contact with the water in the bath, to the feet, which are in contact with a conveyor line. Water bath stunning is applied to relax the neck muscles and contract the wing muscles for easy fixation of the head for automatic killers, prevent excessive struggling and wing flapping during bleed out, facilitate rapid bleeding and relax or loosen feathers [6].

A minimum of 120 mA is required to rupture a chicken's fragile blood vessels. Electroconvulsive treatments of similar amperage given to humans have been reported to produce thunderbolt effects in the heads. This minimum current results in heart failure or immediate death [7]. However, stunning at high current is one of the major factors in deteriorated meat and carcass quality [8,9]. According to Gregory and co-workers [10], 99 % of the birds show ventricular fibrillation with currents greater than 110 mA at 50 Hz. Contreras and Beraquet [11] reported that in a range of 20 to 125 mA, only birds stunned at 100 V, 60 Hz at 125 mA experienced ventricular fibrillation. In recent years, there has been a tendency in Europe to implement high voltage stunning primarily based on humanitarian grounds. When compared to low voltage stunning (in the range of 30 to 60 V, 20 to 45 mA/bird), high voltage stunning (150 V, 100 mA/bird) induces heart fibrillation and cardiac arrest resulting in rapid death [12].

In a study conducted by Contreras and Beraquet [11], the most efficient stunning condition for blood loss was 40 V, which resulted in a 55.3 % blood loss, higher than the 35 to 50 % reported by Newell and Shaffner [13] and the 35 to 50 % blood loss obtained by Potsubay and Duduck [14]. It was also observed that only when the currents used were above 60 mA, 80 V did the birds show symptoms corresponding to the end of respiration, retention of muscular tonus and death. The proportion of birds showing these conditions appeared to be greater for the 100 V treatment; therefore, it was inferred that the reduction in blood loss with higher voltages could be due to a higher incidence of birds with ventricular fibrillation

In general, meat is frequently adulterated, and such irresponsible acts are often veiled by the slaughtering houses. It is relatively easy to differentiate meat to meat contamination because different species of animals carry distinct patterns of genetic makeup. In the stunning and slaughtering technique, the amount of current and voltage delivered to the chickens is hardly detectable in the final product. Modern molecular genomics and the science of genomics have opened up new and exciting possibilities to dissect complex phenotypic traits. These advances have made it possible to develop comprehensive genetic linkage maps in animals such as pigs [15-17]. Mapping the genes of pig indirectly provides a database for identifying meat contaminated with cheaper swine meat. A Real Time quantitative PCR detection method has been developed by Tanabe and colleagues [18] to detect trace amounts of pork, chicken, beef, mutton and horseflesh in food. A number of methods have been developed to detect the meat ingredients in processed foods to verify the labeling. Such methods include the detection of species-specific proteins by enzyme-linked immunosorbent assay (ELISA) and species-specific DNA molecules by polymerase chain reaction (PCR). Currently, species identification is achieved by different methods using protein- and DNA-based assays. Protein-based assays include sodium lauryl sulfate polyacrylamide gel electrophoresis (SDS-PAGE) [19], isoelectric focusing (IEF) [20], ELISA [21] and high pressure liquid chromatography (HPLC) [22]. However, the protein profiles are tissue-dependent, and these proteins may be denatured by processing and heating, with subsequent loss of analytical specificity [23]. Protein techniques also require blotting, long delays to obtain the results, and the preparation of antibodies. Such disadvantages have limited the use of protein techniques in food analysis. However, in cases where the meat origin is similar, such as in the issue of the stunning and slaughtering process, proteomics seems to have advantages over the DNA-based methods. Different currents and voltages are expected to cause changes in protein expression and modification that will be detected by the proteomics approach. Proteins that are differentially expressed depending on the amount of current and voltage used during the stunning process will act as potential biomarkers for identifying the increased electrical heart stimulation.

## Results

**Figure 1:**
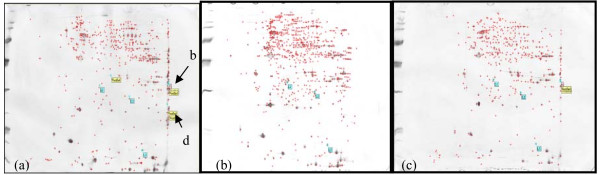
**Protein profiles constructed from the heart samples of chickens stunned with 0.75 A, 70 V (a), or 0.5 A, 40 V (c), or not stunned (0 A, 0 V) (b), respectively**. Each protein spot is represented by a red cross sign on the protein map. The protein map was matched to other maps of different stunning treatments by indicating a few landmarks to ensure correct positioning of the gel maps. Landmark (L1 and L2) are predefined labels displayed on a blue background to mark spots in the gels that serve as reference points. This is for gel alignment or matching, and two annotations are considered to refer to the same point in different gels when they bear identical labels of the same category.

It was observed that in a sample from an animal stunned with 0.75 A, 70 V (Figure 1a), more verified spots were detected in the sample than in the control, suggesting that higher current and voltage induced the overexpression of some proteins in the heart. These proteins were present in the stunned samples but completely absent in the non-stunned samples (0 A, 0 V) (Figure 1b). These proteins were also detected at lower concentrations in a sample from an animal treated with 0.5 A, 40 V (Figure 1c). Therefore, these proteins could be proteins whose expression is influenced by the amount of electrical current and voltage given. To confirm that the identified spots are true biomarkers, scatter plot analysis was performed.

**Figure 2:**
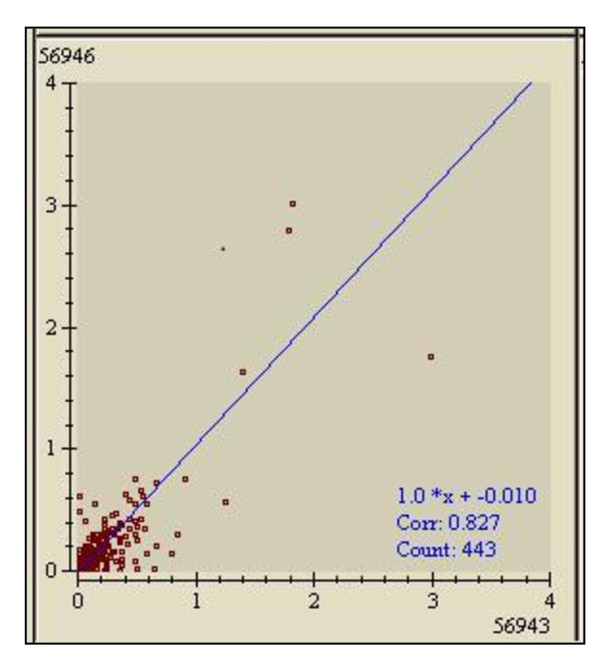
**Scatter plot of heart samples used to analyze gel similarities or experimental variations such as disparities in stain intensity or sample loading for matched spots in sets of gels**. A correlation value close to 1 indicates that the data of the two gels are highly correlated. The count is the number of matched spots displayed. The Y axis displays proteins identified in gel number 56946 (sample from the hearts of chickens stunned with 0.75 A, 70 V), and the X axis is for gel number 56943 (sample from the hearts of non-stunned (0 A, 0 V) chickens).

In Figure 2, for instance, gel number 56946 is displayed on the Y axis and is the reference gel for a chicken stunned with 0.75 A, 70 V. Gel number 56943, which is plotted on the X axis, represents the non-stunned (0 A, 0 V) chicken. The number of matched spots displayed when gel number 56946 is plotted against gel number 56943 is 443, with data correlation of 0.827. Therefore, the two gels are highly correlated. Any new spot emerging on gel number 56946 is considered to be a true biomarker. Based on the scatter plot analysis (Figure 2), two proteins (protein b and d) that were exclusively detected in samples of 0.75 A, 70 V and 0.5 A, 40 V (Figures 1a and 1c) but not in 0 V, 0 A (Figure 1b) are novel biomarkers for electrically stunned chickens.

The results were further verified by performing intraclass analysis (Table 1). Protein b had a mean of 0.3829 with a mean standard deviation of 0.2722 and a variation of 0.7110. The high standard deviation and variation were due to gel-to-gel variation and staining efficiency in the two-dimensional analysis. This gel-to-gel variation could be overcome in the future by employing a fluorescence staining method. However, the identification of these biomarkers is significant because they are not detected at all in non-stunned (0 A, 0 V) samples. The complete absence of these proteins in the controls was confirmed in two biological replicates. The intensities of protein b were determined to be 0.6091 and 0.5394. Therefore, protein b was only detected in two out of three replicates of the 0.75 A, 70 V stunned samples. However, protein b is absent (Figure 1b) in all three replicates of non-stunned samples, making it a protein marker to be considered. In contrast, the mean of the spot intensity for protein d was 0.6047, with a mean standard deviation of 0.1654 and variation of 0.2735. The spot intensities detected on triplicate gels from the 0.75 A, 70 V sample were 0.4094, 0.5908 and 0.8139 (Table 1). Meanwhile, protein d was absent from all the non-stunned samples from three replicates (Figure 1b). Thus, protein b and d were picked from a 2-D gel for MALDI-TOF analysis because they showed consistent patterns of spot intensity related to the current and voltage given in the stunning treatment. Protein b was identified as alpha cardiac muscle actin 1 (ACTC1) and protein d as troponin I by MALDI TOF mass spectrometry (Table 2).

**Table 1:** Intra class report of potential proteins from heart sample

Protein	Mean (100 %)	M.S.D	Variation	Protein Intensity 1	Protein Intensity 2	Protein Intensity 3
b	0.38	0.2722	0.7110	0	0.6091	0.5394
d	0.60	0.1654	0.2735	0.4094	0.5908	0.8139

The intra class report for protein b and protein d are from the triplicate samples of chickens stunned with 0.75 A, 70 V together with the mean standard deviation (M.S.D) and variation.

**Table 2:** MALDI-TOF analysis of potential proteins from heart sample

Spot ID	Protein name	Accession No	Protein Mw (Da)	Protein Score	Protein Score C.I %	Total Ion Score	Total Ion Confirmed C.I %
b	Actin, Alpha Cardiac Muscle 1	ACTC_CHICK	42334	119	100	106	100
d	Troponin I, Cardiac Muscle	TNNI3_CHICK	19081	63	87.5	39	82.4

Proteins were isolated from the heart samples of chickens stunned with 0.75 A, 70 V. The percentage of total ions confirmed gives the confidence level for the identification of a protein spot compared to the protein database.

**Figure 3:**
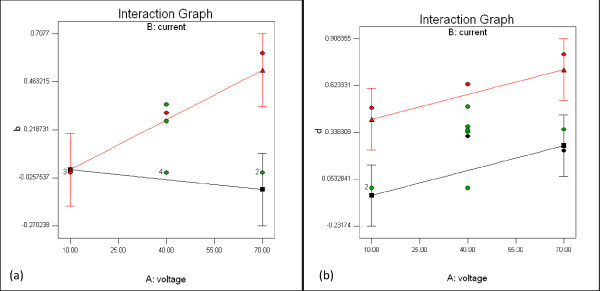
**Interaction graph of **(a) **protein b **(b) **protein d**. The Interaction Graph plots Protein Intensity versus Voltage (V). The red plot represents 0.75 A, while the black plot represents 0.25 A.

**Figure 4:**
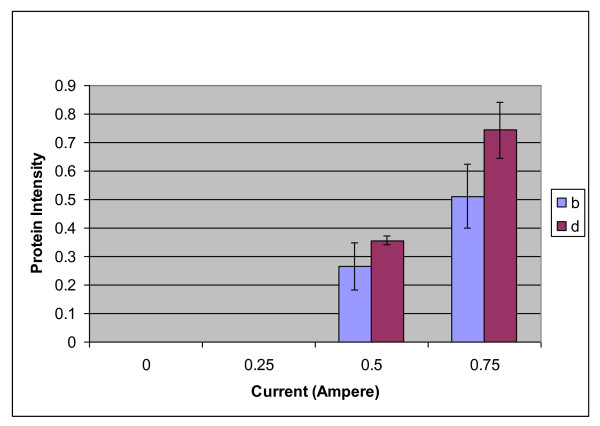
**A bar chart showing protein intensity as a function of the current delivered to the chicken**. Error bars represent the standard deviation of triplicates experiments. Protein b is ACTC1, and d is troponin I.

Interaction graphs were employed to validate the potential proteins identified in the proteomic analysis. Figures 3(a) and 3(b) show the interaction plots of protein b and protein d, respectively. The graphs were generated from the Design-Expert software via 3-level factorial design, and they show that the intensities of protein b and d increased with increasing voltage when the current was maintained at 0.75 A. Meanwhile, when the current applied was 0.25 A, the intensity of protein b decreased, and the intensity of protein d increased. The analysis of variance (ANOVA) shows that for protein b, only the current significantly influenced the protein intensity (p value = 0.0276). For protein d, both the voltage and current showed significant effects (p values = 0.0436 and 0.0054, respectively). Figure 4 shows that alpha cardiac muscle actin 1 (protein b) increased in expression from 0.27 to 0.51 (1.9-fold), while troponin I (protein d) increased from 0.36 to 0.74 (2.1-fold) when the current was increased from 0.5 A to 0.75 A. Interestingly, both verified proteins were completely absent in samples from the non-stunned (0 A) animals and the animals stunned with 0.25 A.

To ensure the reliability of our results, we used Real Time PCR to analyze the expression levels of the corresponding genes in hearts from chickens stunned with 0.75 A, 70 V or non-stunned (0 A, 0 V) (Table 3). The primers for the *ACTC1 *and *TNNI3 *genes were designed (Table 4). *ACTC1 *gene expression was upregulated 142 times in the hearts of chickens stunned with 0.75 A, 70 V, while the *TNNI3 *gene was upregulated by 45 times. The results correlated with findings from intraclass analysis, confirming that both proteins are promising biomarkers for the identification of electrically stunned chickens.

**Table 3:** Real Time PCR of potential proteins from heart sample

Spot ID	Protein Name	Gene Name	Ct Value Sample	Ct Value Control	Ct Value Difference	A value	Fold Increment
b	ACTC1	*ACTC1*	17.0 ± 0.33	17.4 ± 0.35	0.38	-3.258	142.27
d	Troponin I	*Troponin I*	13.5 ± 0.02	13.7 ± 0.03	0.22	-3.374	45.43

The genes are identified as alpha cardiac muscle actin 1 (*ACTC1*) and troponin I (*TNNI3)*. Values are the means of triplicate experiments. "A" value for fold increment was obtained from the equation by Pfaffl, 2001.

**Table 4:** List of primers designed for genes related to potential proteins identified from heart sample

Target gene	Left primer	Right primer	Product size
*ACTC1*	5'-ctgatcgcatgcagaaagaa-3'	5'-acatttgctggaaggtggac-3'	138
*Troponin I*	5'-acgccaagagacaatccaag-3'	5'-gtttctctcctgccctctcc-3'	117

The amplification process was optimized by Polymerase Chain Reaction (PCR) and the conditions used for Real Time PCR.

## Discussions

Alpha cardiac actin is the major component of myofibrils in the cardiac muscle. Actin isoforms (alpha, beta, and gamma) are more than 99 % identical to each other, with small variations in amino acid composition that lead to shifts in the isoelectric points [24-27]. Rai and co-workers [28] have suggested that a high percentage of cardiac actin (approximately 50 %) is arginylated, supporting the possibility that actin arginylation is critical for myofibril integrity. Protein arginylation is a poorly understood posttranslational modification mediated by arginyltransferase [29]. This process involves the transfer of arginine (Arg) from transfer RNA (tRNA) onto proteins [30-32].

Each heartbeat is triggered by a pulse of intracellular calcium ions that bind to troponin on the actin-containing thin filaments of heart muscle cells, initiating a change in filament structure that allows myosin bind and generate force. Sun and co-workers [33] investigated the molecular mechanism of calcium regulation in demembranated trabeculae from rat ventricle using polarized fluorescence from a probe on troponin. Contraction of the heart is mainly driven by an interaction between myosin, actin, and MgATP that is controlled on a beat-to-beat basis by transient binding of Ca^2+ ^ions to the troponin/tropomyosin complex in the actin-containing thin filaments [34-36]. It is clear that troponin and actin interact in the contraction and activation of heart muscle, which is consistent with the findings of this study that the expression of troponin and actin is upregulated in the hearts of chickens stunned with 0.75 A, 70 V.

The activation and relaxation of the heart muscle cells occur over a narrow range of Ca^2+ ^concentrations, and the steep Ca^2+ ^dependence of the regulatory mechanism indicates a high degree of cooperatively. The narrow range of Ca^2+ ^concentrations might be due to coupling between adjacent actin/tropomyosin/troponin units in the thin filament [36-38]. It might also be caused by the activation of the thin filament by myosin binding [39-41].

Hessel and co-workers [42] found that the expression of cardiac troponin I was elevated in the serum of patients without acute coronary syndromes. This response might be due to the release of troponin I from viable cardiomyocytes by stimulation of stretch-responsive integrins. Troponins are myofibrillar proteins involved in the regulation of actin-myosin interaction, thereby controlling the contraction and relaxation of the heart muscle. Cardiac troponins (troponin T, troponin I, and troponin C) are predominantly bound by tropomyosin to actin filaments of sarcomeres, with only a small proportion of troponin T (6-8 %) and troponin I (3-8 %) found in the soluble cytoplasmic pool of the cells [43]. Due to their high cardiac specificity, serum concentrations of troponin I and troponin T are well established diagnostic and prognostic markers of irreversible myocardial damage in acute coronary syndromes [44].

Several studies have reported elevated serum levels of troponins in patients with cardiomyopathy, heart failure, unstable angina pectoris, pulmonary embolism, and renal insufficiency as well as in ultra-endurance athletes. However, there are also cases where elevated serum levels of cardiac troponins have been observed in patients without acute coronary syndromes. Feng and co-workers [45] reported that the degradation of troponin I upon increasing volume and pressure preload might be caused by increased myocardial stretch per second. The mechanical stretch of cardiomyocytes usually occurs during pressure or volume overload. This overloading will cause a cascade of intracellular signals, including increased calcium concentration, increased intracellular nitrogen oxide formation, and the activation of intracellular proteases such as matrix metalloproteinase-2 (MMP2) and MMP14[46,47]. MMP2 is able to degrade troponin I intracellularly and may be involved in the stretch-induced release of troponin I and its degradation products [48]. Therefore, an increased myocardial stretch during the electrical treatment of stunned chickens in this study may be considered to be a pressure overload that results in the release of an elevated amount of troponin I.

## Conclusions

We found alpha cardiac muscle actin 1 and troponin I to be elevated in the samples of hearts of chickens stunned by 0.75 A, 70 V. Both proteins were completely absent in the samples from non-stunned (0 A, 0 V) chickens. Alpha cardiac muscle actin 1 showed an increase in protein intensity from 0.27 to 0.51 when the current was increased from 0.5 A to 0.75 A. Meanwhile, troponin I showed an increase in protein intensity from 0.36 to 0.74 when the current was increased from 0.5 A to 0.75 A. When confirmed at the transcriptional level, the gene coding for actin, alpha cardiac muscle 1 showed upregulation of 142.27-fold in samples from chickens stunned with 0.75 A, 70 V, while the gene for troponin I showed upregulation of 45.43-fold. Therefore, alpha cardiac muscle actin 1 and troponin I are positively expressed in correlation with the current and voltage applied during the stunning treatment.

## Methods

### Experimental Design

Experiments that have been conducted in this project are in compliance with Animal Ethics Guidelines from Department of Veterinary Services and Animals Ordinance, 1953 and this has been approved by the head of Department for Department of Biotechnology Engineering, Faculty of Engineering, IIUM. Female broiler chickens aged 35 days and weighing approximately 1.5 kg were collected from a farm in Kuang, Selangor, Malaysia, electrically stunned, and slaughtered. The two parameters studied for stunning were current and voltage with three levels each: 0.25 A, 0.5 A and 0.75 A for the current and 10 V, 40 V and 70 V for the voltage. Combinations of the two parameters resulted in nine different treatments: (0.25 A, 10 V), (0.25 A, 40 V), (0.25 A, 70 V), (0.5 A, 10 V), (0.5 A, 40 V), (0.5 A, 70 V), (0.75 A, 10 V), (0.75, 40 V) and (0.75, 70 V). These conditions for stunning were compared to the control, which was the non-stunned sample (0 A, 0 V). The amount of current was monitored by an ammeter. The treatments for stunning were designed using a three-level factorial design (Design Expert Software Version 6.0.8). Two biological replicates were studied with three replicates for each sample. A water stunner was designed to supply different sets of current and voltage. A glass aquarium was equipped with a copper plate sized 56.5 cm × 28.5 cm × 28.5 cm. The positive terminal of the circuit was immersed in the copper plate of the aquarium while the negative terminal was attached to the shackle used to hang the chicken's leg. Each chicken was stunned for duration of 5 seconds.

### Sample Preparation

Each heart sample was homogenized in 5 volumes of phosphate buffer (250 mM of PO_4_^+ ^buffer, pH 7.5, 0.01 % Triton X-100). The homogenate was centrifuged at 4°C, 10,000 × *g *for 15 minutes, and the supernatant was retained and stored at -80°C until needed. The protein content of each sample was assayed using the Bradford Assay [49].

### Two-Dimensional Gel Electrophoresis (2-D GE)

Twenty micrograms (20 μg) of sample was added to the sample buffer (7 M urea, 2 M thiourea, 4 % CHAPS, 3 mg/ml DTT, 0.5 % Pharmalyte) resulting in a total volume of 170 μl. Subsequently, 170 μl of rehydration buffer (7 M urea, 2 M thiourea, 4 % CHAPS, trace amount of bromophenol blue, 3 mg/ml DTT, 0.5 % Pharmalyte) was added, and the mixture was left at room temperature for 30 minutes. Isoelectric focusing was conducted on IPGphor 3 according to the manufacturer's protocol (GE Healthcare, Geneva, Switzerland). The temperature was kept constant at 20°C, and the current was maintained at 50 μA per strip. After IEF, a second separation step (SDS-PAGE) was performed on a vertical 12.5 % polyacrylamide gel. Each strip was equilibrated in 10 ml of equilibration buffer (6 M urea, 50 mM Tris-HCl, 30 % glycerol, 2 % SDS). The strips were reduced with 100 mg of DTT dissolved in 10 ml of equilibration buffer and alkylated with 250 mg of iodoacetamide in equilibration buffer with a trace amount of bromophenol blue. The IPG strip was finally rinsed in the same buffer. The IPG strip was sealed in place using 0.5 % agarose solution dissolved by heat in electrophoresis buffer (250 mM Tris, 1.92 M glycine, 1 % SDS). After electrophoresis, the gel was carefully removed from its cassette and stained with silver nitrate solution for gel visualization. After staining was completed, the gels were scanned using ImageScanner III LabScan 6.0 software (GE Healthcare, Geneva, Switzerland). The scanner was set at 300 dpi, transparent mode with green filter.

### Proteomic Analysis of ImageMaster Platinum 6.0

Protein spots were detected and analyzed using ImageMaster 2D Platinum 6.0 software (GE Healthcare, Geneva, Switzerland) to identify the spots of interest. Scatter plot analysis was used to analyze gel similarities or experimental variations such as disparities in stain intensity or sample loading for matched spots in sets of gels. The scatter plot shows the relationship between the spot values, for example, in terms of intensity, volume or percentage volume for two gels by showing the linear dependence between the spot values of one gel on variable X and the corresponding values of the reference gel on the variable Y. After the software analysis, the sample was rerun on the same conditions and stained with Coomassie Brilliant Blue instead of silver nitrate because silver staining is incompatible with mass spectrometry analysis. After staining with Coomassie Brilliant Blue, the spots of interest were manually excised and subjected to MALDI-TOF mass spectrometry analysis. The excised spots from the gel were digested with trypsin and subjected to Zip Tip (Millipore) purification.

### MALDI TOF Mass Spectrometry Analysis

After ZipTip treatment, each spot was analyzed by MALDI TOF/TOF Mass Spectrometer (Applied Biosystems). Both MS and MS/MS spectra were recorded in the combination mode. For the MS spectra, 1800 shots were accumulated for each, and 4500 shots were accumulated for each MS/MS spectrum. Protein was identified from the accumulated MS and MS/MS spectra by manual searching using a locally implemented MASCOT server and was compared to the SwissProt and NCBI database.

### Validation of Proteomics Result by Real Time PCR

To confirm the identified candidate proteins through proteomics analysis, a validation was conducted on the transcriptional level. RNeasy Lipid Tissue Mini Kit (Qiagen) was used to extract total RNA. After the extraction of total RNA, the complementary DNA was synthesized using SuperScript III Cells Direct cDNA Synthesis System (Invitrogen). The single-stranded cDNA concentration was measured, and the sample was stored at -20°C for subsequent PCR amplification. Proteins that had been identified to be positively expressed in relation to the stunning treatment were further studied at the RNA level. The complete sequence for each gene was obtained from the NCBI database. Primers for the candidate biomarkers and a housekeeping gene were designed using Primer3 free software (http://Frodo.wi.mit.edu/primer3/) (Table 4). Real Time PCR was conducted using SYBR Green as the detection method with dissociation curve analysis. A dissociation curve determined whether the amplification is free from contaminants or not. The results were analyzed using MxPro software (Stratagene). The SYBR green Ct values from triplicate experiments were calculated to find an average Ct value for each sample. These average values were then used to calculate the ratio using a formula from Pfaffl [50]. For each experiment, 18S rRNA gene was used as the reference gene, and the non-stunned chicken heart cDNA was the control sample.

## Abbreviations

2D: 2 dimensional; PAGE: Polyacrilamide gel electrophoresis; MALDO-TOF: Matrix assisted laser desorption ionization-time of flight.

## Competing interests

Part of this study is patented under Patent Filing PI 20095435 with the title Method for Determining Amount of Electric Current Delivered to a Fowl Prior to Slaughter.

## Authors' contributions

AA is Chief Investigator who granted with a research grant from the Ministry of Higher Education, the main supervisor for NAS: NAS performed experiments; AA and NAS design the experiment; FY is the co-supervisor for NAS MSc thesis. All authors read and approved the manuscript.
